# PDE1B, a potential biomarker associated with tumor microenvironment and clinical prognostic significance in osteosarcoma

**DOI:** 10.1038/s41598-024-64627-y

**Published:** 2024-06-14

**Authors:** Qingzhong Chen, Chunmiao Xing, Qiaoyun Zhang, Zhijun Du, Jian Kong, Zhongwei Qian

**Affiliations:** 1 Department of Hand Surgery, Affiliated Hospital and Medical School of Nantong University, No.20 West Temple Road, Nantong, 226001 Jiangsu Province China; 2 Nantong University, Nantong, 226001 Jiangsu Province China; 3 Department of Pediatric Surgery, Affiliated Maternity and Child Healthcare Hospital of Nantong University, No.399 Century Avenue, Nantong, 226001 Jiangsu Province China

**Keywords:** PDE1B, Biomarker, Immunity, Osteosarcoma, Prognosis, Data mining, Genome informatics, Cancer genomics, Clinical genetics, Genetic markers

## Abstract

PDE1B had been found to be involved in various diseases, including tumors and non-tumors. However, little was known about the definite role of PDE1B in osteosarcoma. Therefore, we mined public data on osteosarcoma to reveal the prognostic values and immunological roles of the PDE1B gene. Three osteosarcoma-related datasets from online websites were utilized for further data analysis. R 4.3.2 software was utilized to conduct difference analysis, prognostic analysis, gene set enrichment analysis (GSEA), nomogram construction, and immunological evaluations, respectively. Experimental verification of the PDE1B gene in osteosarcoma was conducted by qRT-PCR and western blot, based on the manufacturer's instructions. The PDE1B gene was discovered to be lowly expressed in osteosarcoma, and its low expression was associated with poor OS (all P < 0.05). Experimental verifications by qRT-PCR and western blot results remained consistent (all P < 0.05). Univariate and multivariate Cox regression analyses indicated that the PDE1B gene had independent abilities in predicting OS in the TARGET osteosarcoma dataset (both P < 0.05). GSEA revealed that PDE1B was markedly linked to the calcium, cell cycle, chemokine, JAK STAT, and VEGF pathways. Moreover, PDE1B was found to be markedly associated with immunity (all P < 0.05), and the TIDE algorithm further shed light on that patients with high-PDE1B expression would have a better immune response to immunotherapies than those with low-PDE1B expression, suggesting that the PDE1B gene could prevent immune escape from osteosarcoma. The PDE1B gene was found to be a tumor suppressor gene in osteosarcoma, and its high expression was related to a better OS prognosis, suppressing immune escape from osteosarcoma.

## Introduction

Osteosarcoma, as a common malignancy of the bones, seriously affects children and adolescents aged 15–19 years old, and it is reported to have 4.4 newly diagnosed osteosarcoma cases per million children per year^1,2^. Osteosarcoma occurs commonly in the long bones’ metaphysis, and it is often characterized by poor prognoses, frequent recurrences, as well as distant metastasis properties^3,4^. Currently, treatments for osteosarcoma mainly contain surgery, adjuvant chemotherapy, radiotherapy, drug therapy, immunotherapy, and so on^5^. With these therapies, localized osteosarcoma patients could have five-year survival rates of 60–70%^6^. However, clinical manifestations of early-stage osteosarcoma are less apparent, and more than one in five osteosarcoma patients were diagnosed with metastases, leading to a five-year survival rate of less than 30%^7,8^. Hence, it is urgent to explore the fundamental mechanisms of osteosarcoma and to point out effective prognostic or diagnostic biomarkers for further clinically personalized treatment targets^9^.

Phosphodiesterase 1B (PDE1B) belonged to the phosphodiesterase (PDE) family and PDE1 subfamily, including three main PDE1 isoforms of PDE1A-1C and being stimulated by a calcium-calmodulin complex^10,11^. Zang et al. shed light on that PDE1B had the ability to regulate exosome biogenesis and autophagic flux in microglia for neuroprotection under ischemic stroke conditions^12^. McQuown et al. revealed that PDE1B could serve as a negative regulator of memory in the hippocampus, making it a promising target for memory impairment^13^. In clear cell renal cell carcinoma, PDE1B was found to be the target of miR-5701 in promoting the tumor cells’ apoptosis^14^. Chen et al. established a novel tumor microenvironment (TME)-related signature, including PDE1B, and this signature could predict survival prognosis and therapeutic responses for colon cancer patients^15^. In osteosarcoma, only two gene signatures, including PDE1B, were available, and these two signatures were related to osteosarcoma patients’ overall survival (OS) or metastasis^16,17^. Currently, little was known about the definite role of PDE1B in osteosarcoma. Therefore, the present article was aimed at mining public data on osteosarcoma to reveal the prognostic values and immunological roles of the PDE1B gene in osteosarcoma, with the assistance of experimental verifications. Our results were anticipated to provide operational targets for future clinically personalized treatment targets in osteosarcoma.

## Materials and methods

### Gene screening and data processing

A total of three osteosarcoma-related datasets were utilized for further analysis, containing the GSE28424 osteosarcoma cell line dataset (https://www.ncbi.nlm.nih.gov/geo/query/acc.cgi?acc=GSE28424), including 19 osteosarcoma cells and 4 control cells; the GSE33382 osteosarcoma tissue dataset (https://www.ncbi.nlm.nih.gov/geo/query/acc.cgi?acc=GSE33382), including 84 osteosarcoma tissues and 3 control tissues; and the Therapeutically Applicable Research to Generate Effective Treatments (TARGET) osteosarcoma tissue dataset (https://www.cancer.gov/ccg/research/genome-sequencing/target), including 86 osteosarcoma tissues. With the help of the R “limma” version 3.58.1 package, differential expression analyses were obtained from the GSE28424 and GSE33382 datasets with cut-off values of *P* values below 0.05 for identifying differently expressed genes via the Wilcoxon signed-rank test. By using the R “survival” version 3.5–7 package, survival differences were obtained from the TARGET osteosarcoma dataset with cut-off values of *P* values below 0.05 for identifying OS-significant genes. A Venn diagram was conducted to obtain intersecting genes from these differently expressed and OS-significant genes. Finally, a total of 10 intersected genes were obtained, including the PDE1B gene. Based on our experimental results, the PDE1B gene was selected, and the TARGET osteosarcoma dataset was further utilized for analyzing the roles of the PDE1B gene in osteosarcoma. K-M survival analysis was conducted based on the medium expression of the PDE1B gene, which classified high- and low-risk groups. These analyses were all conducted by the R 4.3.2 software.

### Experimental verifications of PDE1B gene in osteosarcoma

Based on the manufacturer's instructions, quantitative real-time PCR (qRT-PCR) and western blot were conducted to verify the expression levels of the PDE1B gene in osteosarcoma cell lines (MG-63, SW1353, 143B, and hFOB) purchased from Procell (Wuhan, China) using the methods previously described^18,19^. Therein, qRT-PCR was utilized to verify the mRNA expression levels of the PDE1B gene in osteosarcoma cell lines, and the primers were displayed as follows: human-PDE1B-F: 5ʹ-GAGGCTCCATCCGACCAAT-3ʹ, human-PDE1B-R: 5ʹ-GCACCCTTGACCCTAACCC-3ʹ; human-ACTB-F: 5ʹ-TAGTTGCGTTACACCCTTTCTTG-3ʹ, human-ACTB-R 5’-TGCTGTCACCTTCACCGTTC-3ʹ. Western blot was utilized to verify the protein expression levels of the PDE1B gene in osteosarcoma cell lines, and the antibodies were obtained from Abcam (ab182565; https://www.abcam.cn/).

### Associations between PDE1B gene and clinical factors in osteosarcoma

As previously described^20,21^, difference analysis, Cox regression analysis, and nomogram were utilized to reveal the associations between the PDE1B gene and clinical factors in osteosarcoma, respectively. Therein, boxplots were applied to display the PDE1B gene expression distribution in different clinical factors (gender of female and male; race of African, Asian, and White; first event of relapse, censored or none; disease at diagnosis of metastatic or nonmetastatic; primary tumor site of arm/hand/pelvis, and leg/foot). Moreover, it is statistically called censored if the outcome event does not occur at the specified end time due to loss of visit, death, failure to recover, etc. Univariate and multivariate Cox regression analyses were utilized to find clinical factors with independent abilities in predicting OS for osteosarcoma. A nomogram was also put in place to intuitively forecast the survival probabilities of osteosarcoma patients. C-index, ROC curves, and calibration plots were utilized to assess the performance of the established nomogram in osteosarcoma. These analyses were all conducted by the R 4.3.2 software.

### Pathway predictions and immunological associations

To obtain PDE1B related pathways in osteosarcoma, gene set enrichment analysis (GSEA) was applied in high-PDE1B and low-PDE1B subgroups by the GSEA 4.0.0 software with default parameters as previously described^22,23^. To reveal the associations between PDE1B gene expression and immunity in osteosarcoma, tumor immune cells infiltration levels and tumor microenvironment were assessed based on the medium expression of the PDE1B gene, classified into high- and low-risk groups. Tumor immune cells infiltration levels were calculated by the “CIBERSORT” version 1.03 R script to assess the infiltration levels of 22 kinds of immune cells in high-PDE1B and low-PDE1B subgroups^24,25^. Tumor microenvironment was calculated by the “ESTIMATE” algorithm of the R “estimate” version 1.0.13 package to assess immune, ESTIMATE, and stromal scores in high-PDE1B and low-PDE1B subgroups^26,27^. The Tumor Immune Dysfunction and Exclusion website (TIDE; http://tide.dfci.harvard.edu/) with default parameters was also applied to forecast immune responses by evaluating patients’ PDE1B gene expression to immunotherapies^28,29^. These analyses were conducted by the R 4.3.2 software.

### Ethics approval and consent to participate

This study was approved by the Institutional Research Ethics Committees of Affiliated Hospital of Nantong University.

## Results

### The expression of PDE1B in osteosarcoma

**Figure 1:**
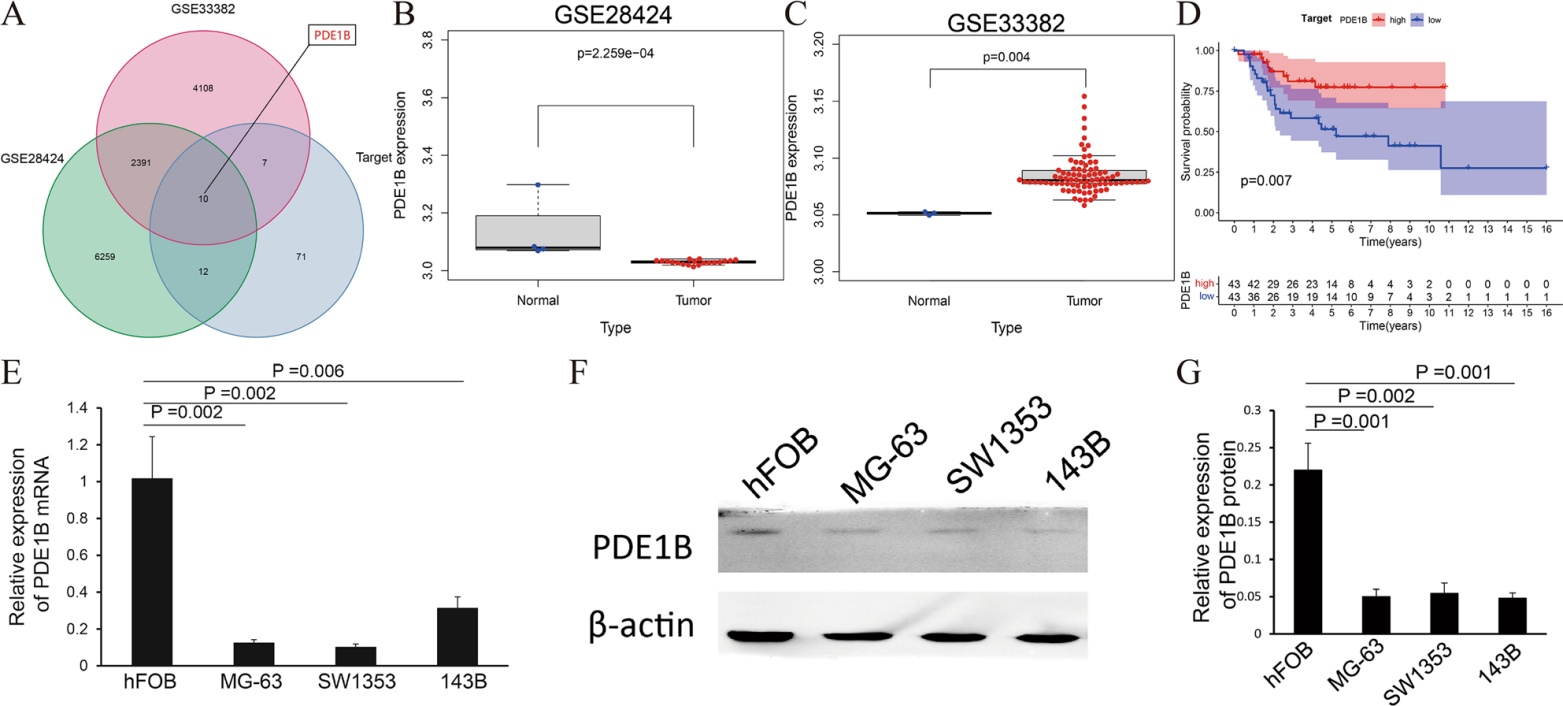
The expression of PDE1B in osteosarcoma; (**A**) Venn diagram for gene filtration; (**B**) PDE1B gene expression in GSE28424 dataset; (**C**) PDE1B gene expression in GSE33382 dataset; (**D**) PDE1B gene survival analysis in TARGET osteosarcoma dataset; (**E**) qRT-PCR results of PDE1B gene in osteosarcoma cell lines; (**F**) Western blot results of PDE1B gene in osteosarcoma cell lines; (**G**) Bar chart of western blot results.

The whole analyzing process was detailed in Fig. S1. The Venn diagram showed that a total of 10 intersected genes were obtained from three osteosarcoma-related datasets (GSE28424, GSE33382, and TARGET osteosarcoma datasets), including the PDE1B gene (Fig. 1A). Therein, PDE1B was differently expressed in the GSE28424 and GSE33382 datasets, with the cut-off values of the P value below 0.05 (Fig. 1B,C). Moreover, PDE1B was significantly associated with OS in the TARGET osteosarcoma dataset, with a cut-off value of P value below 0.05 (Fig. 1D). Experimental verifications by qRT-PCR and western blot results indicated that both PDE1B gene mRNA and protein expression levels were lowly expressed in osteosarcoma cell lines (all P < 0.05; Fig. 1E–G). The above-mentioned results showed that PDE1B was lowly expressed in osteosarcoma and significantly associated with OS.

### Clinical factors’ relationships with PDE1B gene in osteosarcoma

**Figure 2:**
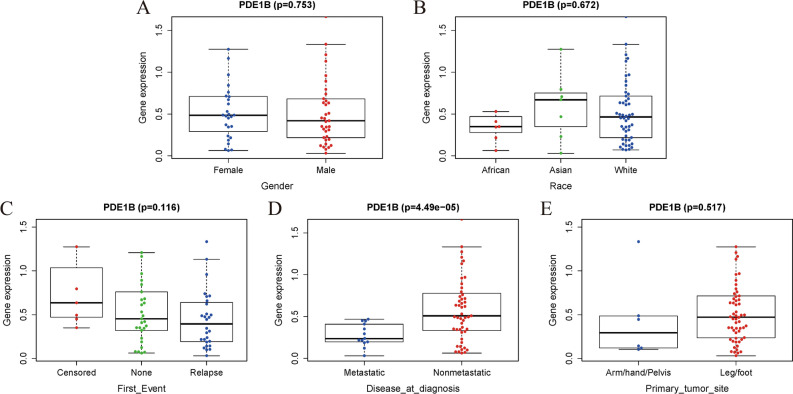
Boxplots of PDE1B gene expression distribution in (**A**) gender of female and male; (**B**) race of African, Asian, and White; (**C**) first event of relapse, censored or none; (**D**) disease at diagnosis of metastatic or nonmetastatic; (**E**) primary tumor site of arm/hand/pelvis, and leg/foot.

**Figure 3:**
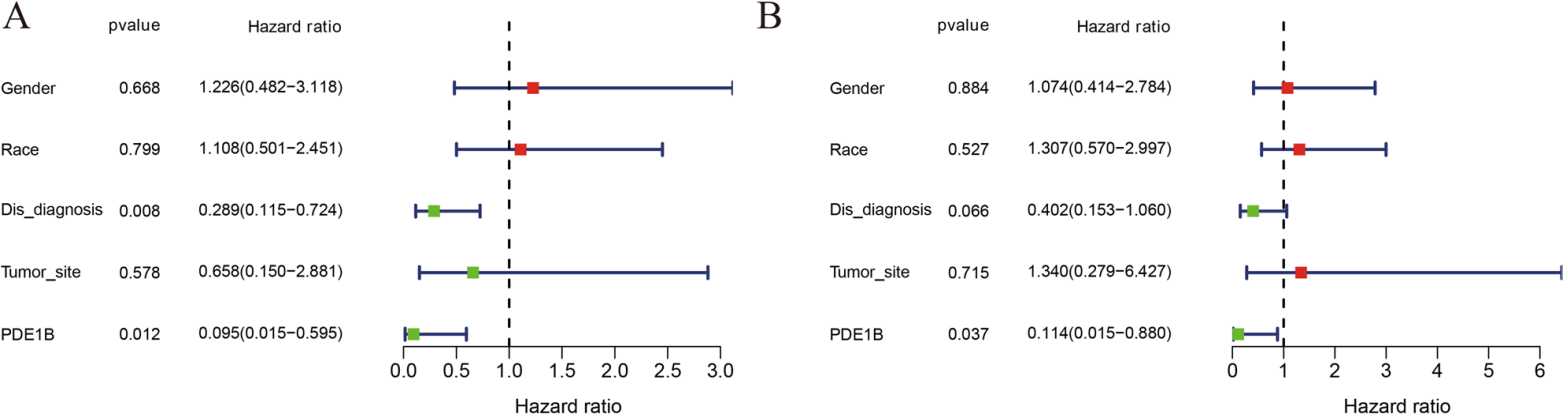
Univariate and multivariate cox regression analyses; (**A**) Univariate cox regression analyses; (**B**) Multivariate cox regression analyses.

**Figure 4:**
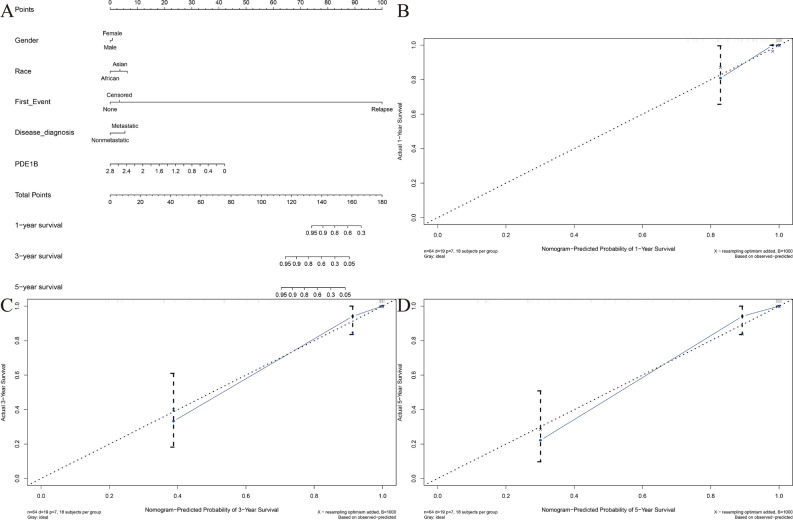
Nomogram and its evaluations; (**A**) Nomogram; (**B**) 1-year calibration plot; (**C**) 3-year calibration plot; (**D**) 5-year calibration plot.

Figure 2 presented the PDE1B gene expression distribution in different clinical factors (gender, race, first event, disease at diagnosis, and primary tumor site). Our results indicated that the PDE1B gene was remarkably related to metastasis (P < 0.001; Fig. 2D) in the TARGET osteosarcoma dataset, while it was not associated with gender, race, first event, or primary tumor site (all P > 0.05). Univariate and multivariate Cox regression analyses indicated that the PDE1B gene had independent abilities in predicting OS in the TARGET osteosarcoma dataset (both P < 0.05; Fig. 3 and Table 1). To further forecast the 1-, 3-, 5-year OS probabilities of osteosarcoma patients intuitively, a nomogram was established based on several clinical factors (gender, race, first event, disease at diagnosis, and PDE1B gene expression) (Fig. 4A). C-index and 1-, 3-, 5-year AUCs of the PDE1B-based nomogram were 0.867, 0.892, 0.899, and 0.873 in the Target osteosarcoma dataset, respectively (Table 2). 1-, 3-, 5-year calibration plots indicated the consistency between actual and ideal results, suggesting a good performance of our established nomogram in osteosarcoma (Fig. 4B–D). The above-mentioned results showed that PDE1B was significantly associated with metastasis and had independent abilities in predicting OS in osteosarcoma.

**Table 1:** Prediction of overall survival by using univariate and multivariate analyses of PDE1B expression level and clinicopathological variables in Target osteosarcoma dataset.

Variables	Univariate analysis				Multivariate analysis			
	HR	HR.95L	HR.95H	pvalue	HR	HR.95L	HR.95H	pvalue
Gender	1.226386	0.482331	3.118234	0.66821	1.073713	0.414106	2.783972	0.883677
Race	1.108442	0.501196	2.451423	0.799314	1.306925	0.56989	2.997162	0.52732
Dis_diagnosis	0.288871	0.115323	0.723585	**0.008036**	0.402376	0.152714	1.060194	0.065517
Tumor_site	0.657825	0.150216	2.880754	0.578338	1.339855	0.279311	6.427276	0.714591
PDE1B	0.094505	0.015019	0.59465	**0.011942**	0.114401	0.014876	0.879791	**0.03725**

Bold font means P < 0.05.

*HR* hazard ratio, *HR.95L* lower limit of HR 95% confidence interval, *HR.95H* upper limit of HR 95% confidence interval, *Gender* female or male, *Race* African, Asian, or White; *Dis_diagnosis* metastatic or nonmetastatic, *Tumor_site* arm/hand/pelvis or leg/foot.

**Table 2:** C-index and 1-, 3-, 5-year AUCs of PDE1B based nomogram in Target osteosarcoma dataset.

	1-year	3-year	5-year	C-index
AUC	0.867	0.892	0.899	0.873

### PDE1B related pathways and its associations with immune checkpoints genes, m6A genes, VEGF pathway genes in osteosarcoma

**Figure 5:**
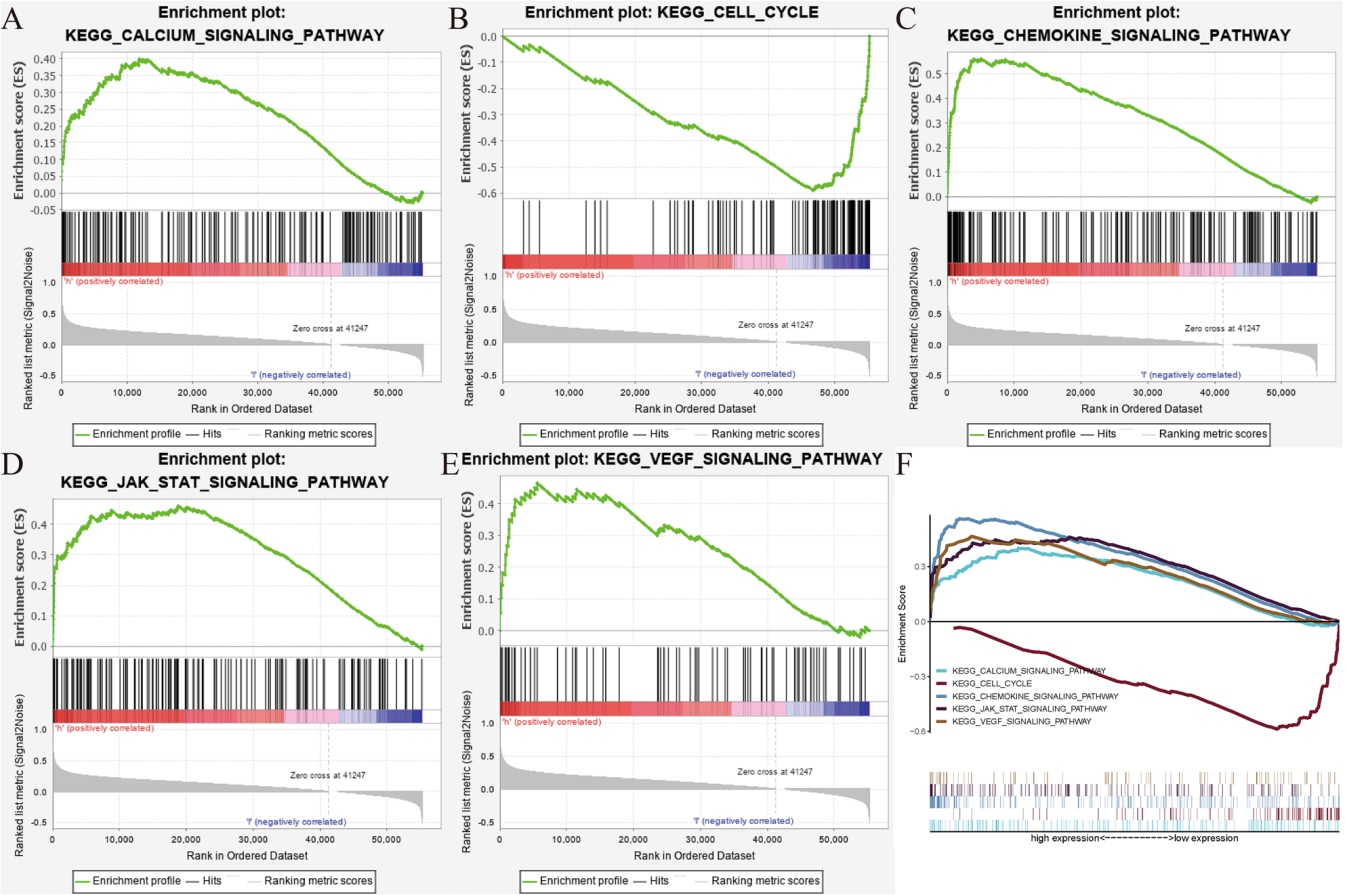
PDE1B-related signaling pathways; (**A**) Calcium pathway; (**B**) Cell cycle pathway; (**C**) Chemokine pathway; (**D**) JAK STAT pathway; (**E**) VEGF pathway; (**F**) All of these five pathways.

**Figure 6:**
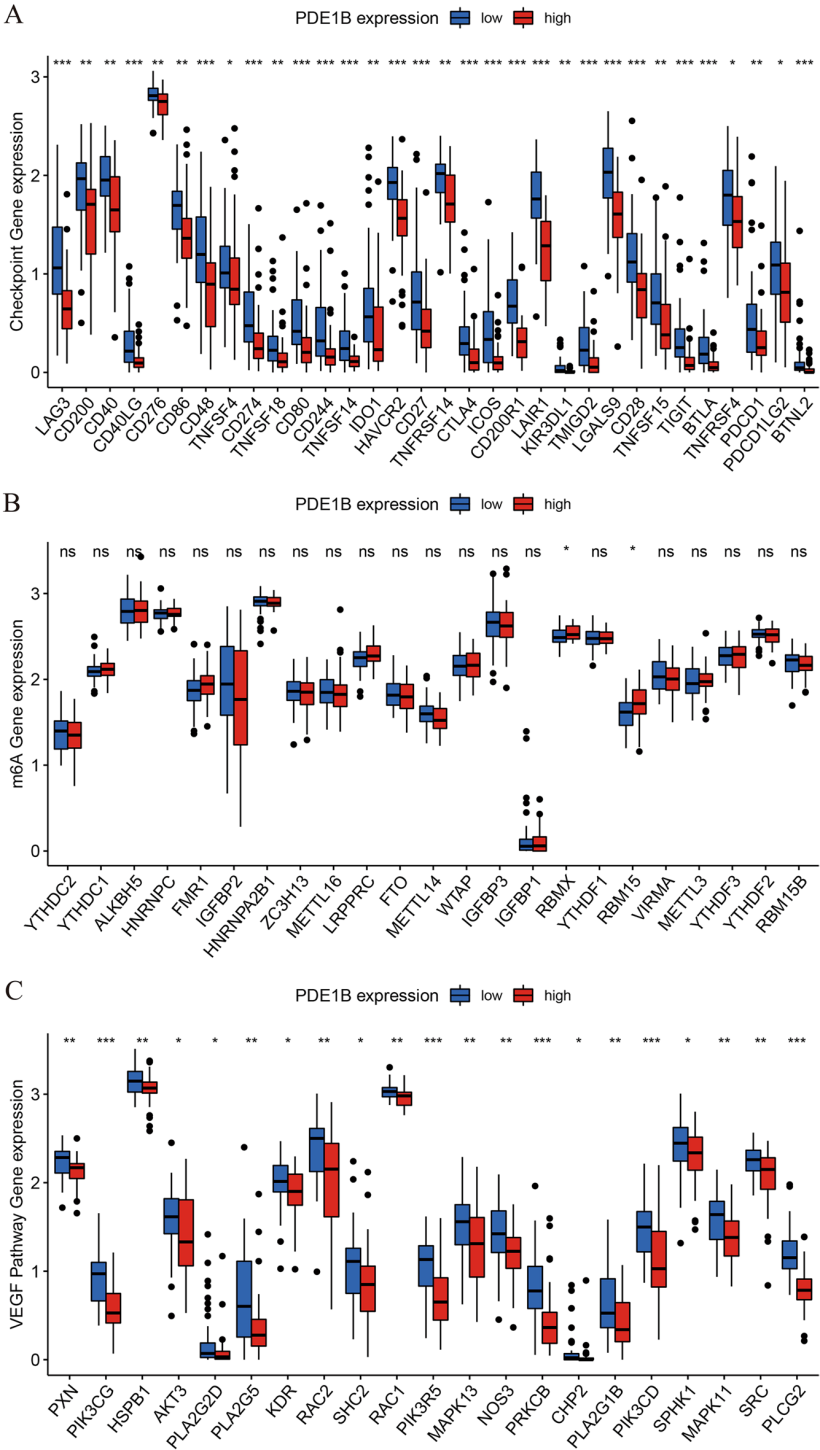
Correlations between PDE1B expression and (**A**) immune checkpoints genes; (**B**) m6A regulator genes; (**C**) VEGF pathway genes; *P < 0.05; **P < 0.01; ***P < 0.001; *ns* not significant.

GSEA was applied by us to reveal PDE1B related pathways in osteosarcoma, and our outcomes showed that PDE1B was markedly linked to calcium, cell cycle, chemokine, JAK STAT, and VEGF pathways (Fig. 5 and Table 3). We further explored the immune checkpoint gene, m6A gene, and VEGF pathway gene expression levels in low- and high-PDE1B subgroups (Fig. 6). Our results reported that the PDE1B gene was significantly involved with immune checkpoint genes and VEGF pathway genes. For m6A regulator genes, only RBMX and RBM15 were firmly related to PDE1B gene expression. The above-mentioned results showed that PDE1B was significantly associated with five pathways, immune checkpoint genes and VEGF pathway genes in osteosarcoma.

**Table 3:** Gene sets enrichment analysis of PDE1B mRNA expression in Target osteosarcoma dataset.

Gene set name	NES	NOM p-val	FDR q-val
KEGG_CALCIUM_SIGNALING_PATHWAY	1.712	0.010	0.101
KEGG_CELL_CYCLE	-2.024	0.015	0.089
KEGG_CHEMOKINE_SIGNALING_PATHWAY	2.093	< 0.001	0.024
KEGG_JAK_STAT_SIGNALING_PATHWAY	1.812	0.004	0.063
KEGG_VEGF_SIGNALING_PATHWAY	1.961	0.002	0.035

*NES* normalized enrichment score, *NOM* nominal, *FDR* false discovery rate.

### Associations between PDE1B gene expression and immunity in osteosarcoma

**Figure 7:**
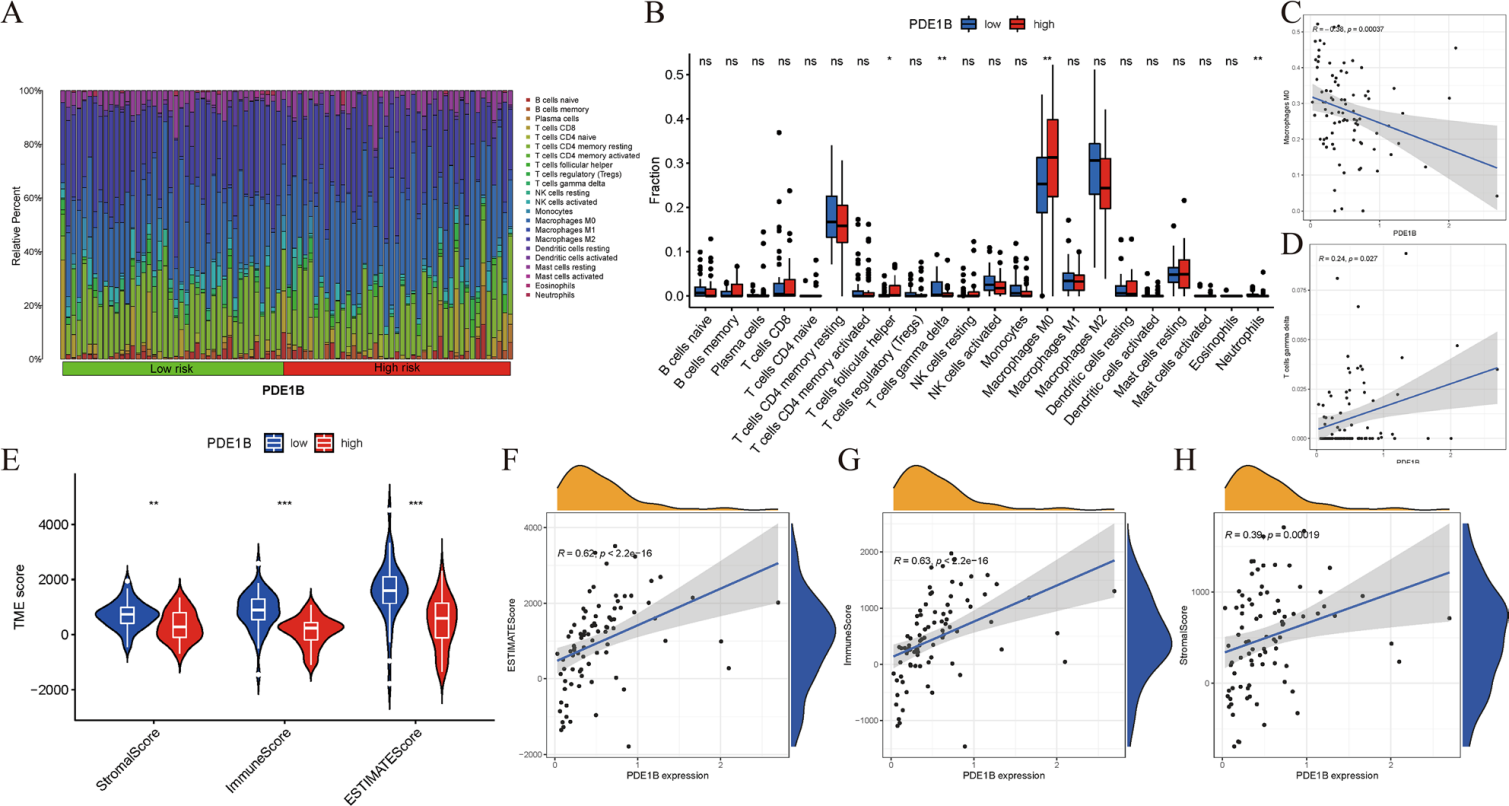
Correlations between PDE1B expression and immunity in osteosarcoma; (**A**) Proportions of tumor immune cells infiltration levels in tissues of TARGET osteosarcoma dataset; (**B**) 22 kinds of immune cells in high-PDE1B and low-PDE1B subgroups; (**C**) Correlation analyses between PDE1B and Macrophages M0 cells infiltration levels; (**D**) Correlation analyses between PDE1B and T cells gamma delta infiltration levels; (**E**) Tumor microenvironment evaluations; (**F**) Correlation analyses between PDE1B and ESTIMATE score; (**G**) Correlation analyses between PDE1B and immune score; (**H**) Correlation analyses between PDE1B and stromal score; *P < 0.05; **P < 0.01; ***P < 0.001; *ns* not significant.

To reveal the associations between PDE1B gene expression and immunity in osteosarcoma, tumor immune cells infiltration levels and tumor microenvironment were assessed based on the medium expression of the PDE1B gene, classified into high- and low-risk groups. Tumor immune cells infiltration levels were calculated by the CIBERSORT algorithm to assess the infiltration levels of 22 kinds of immune cells in high-PDE1B and low-PDE1B subgroups (Fig. 7A). The infiltration levels of T cells follicular helper, T cells gamma delta, Macrophages M0, and Neutrophils were differently expressed in high-PDE1B and low-PDE1B subgroups (all P < 0.05; Fig. 7B). Further correlation analyses showed that PDE1B gene expression was significantly associated with T cells gamma delta or Macrophages M0 cells infiltration levels (all P < 0.05; Fig. 7C,D). The tumor microenvironment was calculated by the ESTIMATE algorithm to assess immune, ESTIMATE, and stromal scores in high-PDE1B and low-PDE1B subgroups. Our results presented that immune, ESTIMATE, and stromal scores were differently expressed in high-PDE1B and low-PDE1B subgroups (all P < 0.05; Fig. 7E). Moreover, correlation analyses showed that PDE1B gene expression was significantly associated with immune, ESTIMATE, and stromal scores (all P < 0.05; Fig. 7F–H). The above-mentioned results showed that PDE1B was significantly associated with immunity in osteosarcoma.

### Evaluating osteosarcoma patients’ PDE1B gene expression for immunotherapies by TIDE dataset.

**Figure 8:**
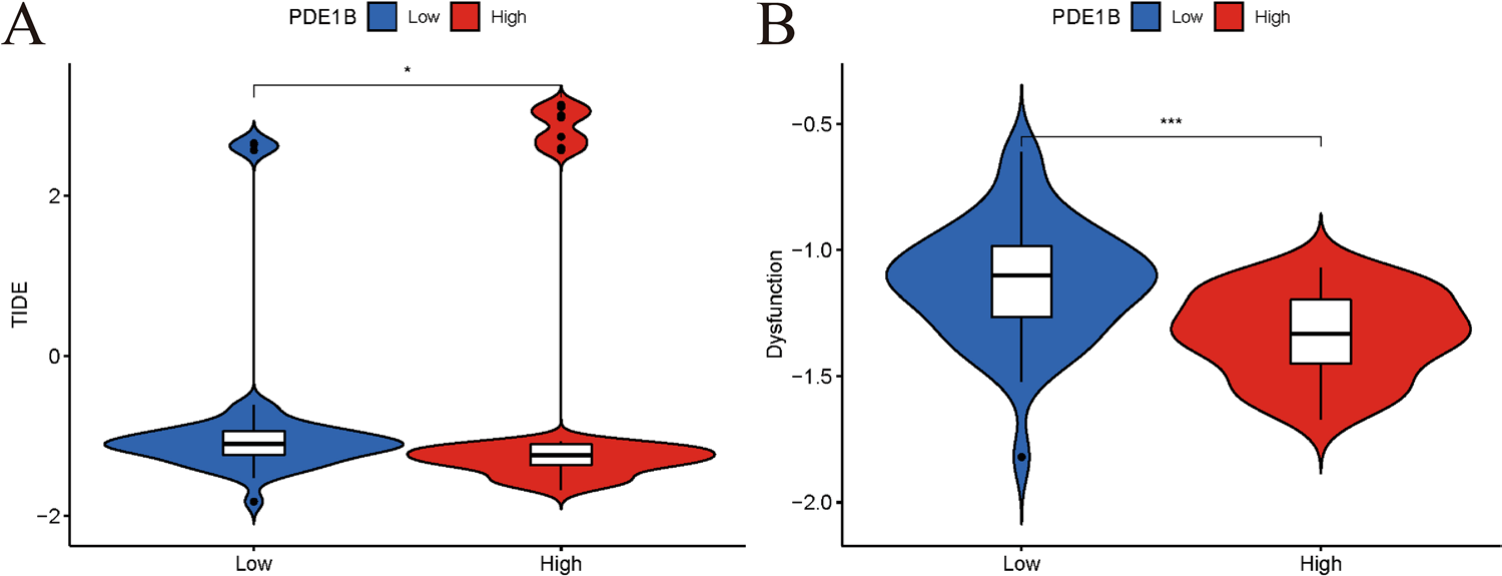
Evaluating osteosarcoma patients’ PDE1B gene expression for immunotherapies by TIDE dataset; (**A**) TIDE scores in high-PDE1B and low-PDE1B subgroups; (**B**) T cell dysfunction scores in high-PDE1B and low-PDE1B subgroup; *P < 0.05;***P < 0.001.

The TIDE algorithm was also applied to forecast immune responses by evaluating patients’ PDE1B gene expression for immunotherapies. The outcomes of us shed light on that patients with high-PDE1B expression would have a lower TIDE score and a lower T cell dysfunction score (Fig. 8). The above-mentioned results showed that patients with high-PDE1B expression would have a better immune response to immunotherapies than those with low-PDE1B expression in osteosarcoma.

## Discussion

Osteosarcoma was characterized by poor prognoses, frequent recurrences, as well as distant metastasis properties, seriously affecting children and adolescents aged 15–19 years old^30^. Despite the progress in treatments for osteosarcoma containing surgery, adjuvant chemotherapy, radiotherapy, drug therapy, immunotherapy, and so on, more than one in five osteosarcoma patients were diagnosed with metastases, leading to a five-year survival rate of less than 30%^31^. The PDE1B gene belonged to the PDE family, and this gene had been found to be involved in various diseases, including tumors and non-tumors. Currently, little was known about the definite role of PDE1B in osteosarcoma. Therefore, we mined public data on osteosarcoma to reveal the prognostic values and immunological roles of the PDE1B gene, providing operational targets for further clinically personalized treatment targets in osteosarcoma.

In this article, we firstly mined three osteosarcoma related datasets (GSE28424, GSE33382 and TARGET osteosarcoma datasets) and identified a total of 10 intersected genes by Venn diagram, including the PDE1B gene. As reported, Tan et al. identified and confirmed the roles of ABCG8, PDE1B, and LOXL4 in osteosarcoma for predicting OS^16^. Wen et al. also found a three-gene signature, including MYOM2, PDE1B, and COCH genes, that could predict the OS of osteosarcoma^17^. In our article, the PDE1B gene was discovered to be lowly expressed in osteosarcoma, and its low expression was significantly associated with poor OS. Experimental verifications by qRT-PCR and western blot results remained consistent. In the associations with clinical factors, the PDE1B gene was remarkably related to metastasis. Univariate and multivariate Cox regression analyses indicated that the PDE1B gene had independent abilities in predicting OS in the TARGET osteosarcoma dataset. We also established a nomogram based on several clinical factors (gender, race, first event, disease at diagnosis, and PDE1B gene expression) to further forecast the 1-, 3-, 5-year OS probabilities of osteosarcoma patients intuitively with good performance. GSEA revealed that PDE1B was markedly linked to the calcium, cell cycle, chemokine, JAK STAT, and VEGF pathways. Moreover, PDE1B was found to be markedly associated with immunity, including the T cells gamma delta, Macrophages M0 cell infiltration levels, and the tumor microenvironment. Further TIDE algorithms shed light on that patients with high-PDE1B expression would have a better immune response to immunotherapies than those with low-PDE1B expression in osteosarcoma.

GSEA was applied by us to reveal PDE1B related pathways in osteosarcoma, and the outcomes of us showed that PDE1B was markedly linked to the calcium, cell cycle, chemokine, JAK STAT, and VEGF pathways. Previously articles showed that calcium homeostasis could inhibit cell proliferation and migration regulated by melatonin^32^. Zheng et al. identified POGZ as a hub gene in osteosarcoma, related to the cell cycle pathway^33^. Fan et al. found that dihydrotanshinone I could regulate osteosarcoma U-2 OS cells’ adhesion and migration via chemokine pathways^34^. As for the JAK STAT pathway, Lv et al. utilized bioinformatics analyses and firstly mined serglycin as a prognostic marker involved with the JAK/STAT pathway^35^. Assi et al. summarized the roles of VEGF signaling pathways in osteosarcoma, pointing out future directions^36^. All of these five signaling pathways were significantly linked with osteosarcoma, and the PDE1B gene was predicted to play vital roles via these pathways in osteosarcoma.

To reveal the associations between PDE1B gene expression and immunity in osteosarcoma, immune checkpoints genes, tumor immune cells infiltration levels and tumor microenvironment were conducted based on the medium expression of the PDE1B gene, classified into high- and low-risk groups. As for immune checkpoints genes, our results reported that the PDE1B gene was significantly involved with immune checkpoints genes in osteosarcoma. Gul Mohammad et al. established an RBP-based model and found significant associations between this model and immune checkpoint genes, providing biomarkers to predict potential immune responses in osteosarcoma^37^. In the case of tumor immune cells infiltration levels, PDE1B was found to be markedly associated with T cells gamma delta and Macrophages M0 cells infiltration levels. As reported, Niu et al. found that Macrophages M0 and M2 cells were the major infiltrated cells in osteosarcoma by the CIBERSORT algorithm^38^. Wang et al. reported that the TUBB1 gene was positively linked to T cells gamma delta infiltration levels in osteosarcoma^39^. In terms of tumor microenvironment, our results presented that PDE1B gene expression was significantly associated with immune, ESTIMATE, and stromal scores by the ESTIMATE algorithm. Likewise in other published articles, Ma et al. successfully established an autophagy-related signature for risk stratification, survival prediction, and tumor microenvironment evaluation in osteosarcoma^40^. He et al. successfully constructed an immune-based LncRNA model to forecast osteosarcoma patients’ prognosis and to evaluate the tumor microenvironment^41^. All of these indicated the vital associations between PDE1B gene expression and immunity in osteosarcoma.

However, no PDE1B targeted treatments were available currently. So, the TIDE algorithm was also applied by us to forecast immune responses by evaluating patients’ PDE1B gene expression for immunotherapies for further clinical evaluations. The TIDE algorithm had been widely used in other studies for predicting immune responses. Wang et al. identified a ferroptosis-related model and applied the TIDE algorithm to show that low-risk patients could benefit more from immunotherapies for lung squamous cell carcinoma^42^. Zhou et al. established a pyroptosis-based LncRNA model and also calculated the TIDE algorithm to forecast immune responses of their signature in kidney cancer^43^. In the present article, the outcomes of us shed light on that patients with high-PDE1B expression would have a lower TIDE score and a lower T cell dysfunction score. In other words, patients with high-PDE1B expression would have a better immune response to immunotherapies than those with low-PDE1B expression. All of these suggested that elevated PDE1B gene expression could prevent immune escape from osteosarcoma.

Future research would focus on using advanced computational models to identify novel mRNA or miRNA biomarkers for complex human diseases, which was crucial for identifying biomarkers in diseases like osteosarcoma. Recent studies had demonstrated that integrating various data sources and algorithms could significantly improve predictive accuracy^44–46^. For instance, the Rotation Forest for Essential MicroRNA Identification (RFEM), which combined multiple miRNA functional features, had shown notable improvements in miRNA identification accuracy^47^. These developments indicated that computational approaches utilizing data integration and multi-model strategies were vital for future mRNA or miRNA biomarker research. In our subsequent studies, we would further mine hub mRNA or miRNA biomarkers for osteosarcoma via various advanced computational models.

In this article, we firstly mined the prognostic values and immunological roles of the PDE1B gene in osteosarcoma with the assistance of experimental verifications, making our results much more reliable. This article provided some references for future PDE1B related research in osteosarcoma and was anticipated to provide operational targets for future clinically personalized treatment targets for osteosarcoma. The major limitation was that the current paper was only a preliminary exploration of the role of PDE1B in osteosarcoma, which was more based on theoretical analysis. Further in-depth in vitro and in vivo experiments in our subsequent articles were still required to verify these findings.

## Conclusions

The PDE1B gene was found to be a tumor suppressor gene in osteosarcoma, related to OS prognosis and immunity. Univariate and multivariate Cox regression analyses indicated that the PDE1B gene had independent abilities in predicting OS for osteosarcoma. GSEA revealed that PDE1B was markedly linked to the calcium, cell cycle, chemokine, JAK STAT, and VEGF pathways. Further TIDE algorithms shed light on that patients with high-PDE1B expression would have a better immune response to immunotherapies than those with low-PDE1B expression, suggesting that elevated PDE1B gene expression could prevent immune escape from osteosarcoma. Further in-depth in vitro and in vivo experiments were still required to verify our findings.

### Supplementary Information

Supplementary Information 1.

## Data Availability

The RNA-sequencing data and corresponding clinical information were downloaded from Therapeutically Applicable Research to Generate Effective Treatments (TARGET; https://ocg.cancer.gov/programs/target) osteosarcoma tissue dataset. All data used to support the findings of this study were included within the article. Please contact corresponding author for data requests.

## References

[1] Mirabello L, Troisi RJ, Savage SA (2009). Osteosarcoma incidence and survival rates from 1973 to 2004: Data from the Surveillance, Epidemiology, and End Results Program. Cancer.

[2] Eaton BR, Schwarz R, Vatner R, Yeh B, Claude L, Indelicato DJ, Laack N (2021). Osteosarcoma. Pediatr. Blood Cancer.

[3] Liao J, Han R, Wu Y, Qian Z (2021). Review of a new bone tumor therapy strategy based on bifunctional biomaterials. Bone Res..

[4] Chen C, Xie L, Ren T, Huang Y, Xu J, Guo W (2021). Immunotherapy for osteosarcoma: Fundamental mechanism, rationale, and recent breakthroughs. Cancer Lett..

[5] Xu K, Fei W, Huo Z, Wang S, Li Y, Yang G, Hong Y (2022). PDCD10 promotes proliferation, migration, and invasion of osteosarcoma by inhibiting apoptosis and activating EMT pathway. Cancer Med..

[6] Zhang Y, He R, Lei X, Mao L, Yin Z, Zhong X, Cao W, Zheng Q, Li D (2022). Comprehensive analysis of a ferroptosis-related lncRNA signature for predicting prognosis and immune landscape in osteosarcoma. Front. Oncol..

[7] Brion R, Regnier L, Mullard M, Amiaud J, Rédini F, Verrecchia F (2021). LIM kinases in osteosarcoma development. Cells.

[8] Wang Y, Wang X, Su X, Liu T (2017). HIF-2α affects proliferation and apoptosis of MG-63 osteosarcoma cells through MAPK signaling. Mol. Med. Rep..

[9] Zeng W, Xu H, Wei T, Liang H, Ma X, Wang F (2022). Overexpression of BRINP3 predicts poor prognosis and promotes cancer cell proliferation and migration via MAP4 in osteosarcoma. Dis. Mark..

[10] Siuciak JA, McCarthy SA, Chapin DS, Reed TM, Vorhees CV, Repaske DR (2007). Behavioral and neurochemical characterization of mice deficient in the phosphodiesterase-1B (PDE1B) enzyme. Neuropharmacology.

[11] Reed TM, Browning JE, Blough RI, Vorhees CV, Repaske DR (1998). Genomic structure and chromosome location of the murine PDE1B phosphodiesterase gene. Mammal. Genome.

[12] Zang J, Wu Y, Su X, Zhang T, Tang X, Ma D, Li Y, Liu Y, Weng Z, Liu X (2020). Inhibition of PDE1-B by vinpocetine regulates microglial exosomes and polarization through enhancing autophagic flux for neuroprotection against ischemic stroke. Front. Cell Dev. Biol..

[13] McQuown S, Xia S, Baumgärtel K, Barido R, Anderson G, Dyck B, Scott R, Peters M (2019). Phosphodiesterase 1b (PDE1B) regulates spatial and contextual memory in hippocampus. Front. Mol. Neurosci..

[14] Zhao C, Mo L, Lei T, Yan Y, Han S, Miao J, Gao Y, Wang X, Zhao W, Huang C (2021). miR-5701 promoted apoptosis of clear cell renal cell carcinoma cells by targeting phosphodiesterase-1B. Anti-cancer Drugs.

[15] Chen W, Huang J, Xiong J, Fu P, Chen C, Liu Y, Li Z, Jie Z, Cao Y (2021). Identification of a tumor microenvironment-related gene signature indicative of disease prognosis and treatment response in colon cancer. Oxid. Med. Cell. Longev..

[16] Tan J, Liang H, Yang B, Zhu S, Wu G, Li L, Liu Z, Li L, Qi W, Li S (2021). Identification and analysis of three hub prognostic genes related to osteosarcoma metastasis. J. Oncol..

[17] Wen C, Wang H, Wang H, Mo H, Zhong W, Tang J, Lu Y, Zhou W, Tan A, Liu Y (2020). A three-gene signature based on tumour microenvironment predicts overall survival of osteosarcoma in adolescents and young adults. Aging.

[18] Ma F, Li X, Fang H, Jin Y, Sun Q, Li X (2020). Prognostic value of ANXA8 in gastric carcinoma. J. Cancer.

[19] Cai X, Qiu W, Qian M, Feng S, Peng C, Zhang J, Wang Y, Wang Y (2020). A Candidate prognostic biomarker complement factor I promotes malignant progression in glioma. Front. Cell Dev. Biol..

[20] Zhengqi Q, Zezhi G, Lei J, He Q, Jinyao P, Ying A (2021). Prognostic role of PHYH for overall survival (OS) in clear cell renal cell carcinoma (ccRCC). Eur. J. Med. Res..

[21] Li X, Yu W, Liang C, Xu Y, Zhang M, Ding X, Cai X (2020). INHBA is a prognostic predictor for patients with colon adenocarcinoma. BMC Cancer.

[22] Subramanian A, Tamayo P, Mootha VK, Mukherjee S, Ebert BL, Gillette MA, Paulovich A, Pomeroy SL, Golub TR, Lander ES (2005). Gene set enrichment analysis: A knowledge-based approach for interpreting genome-wide expression profiles. Proc. Natl. Acad. Sci. USA.

[23] Wang Y, Liu S, Chen Y, Zhu B, Xing Q (2022). Survival prognosis, tumor immune landscape, and immune responses of PPP1R18 in kidney renal clear cell carcinoma and its potentially double mechanisms. World J. Oncol..

[24] Gao Y, Li JY, Mao JY, Zhou JF, Jiang L, Li XP (2022). Comprehensive analysis of CRIP1 expression in acute myeloid leukemia. Front. Genet..

[25] Zhang S, Zhang W, Zhang J (2022). 8-Gene signature related to CD8(+) T cell infiltration by integrating single-cell and bulk RNA-sequencing in head and neck squamous cell carcinoma. Front. Genet..

[26] Maimaiti A, Tuersunniyazi A, Meng X, Pei Y, Ji W, Feng Z, Jiang L, Wang Z, Kasimu M, Wang Y (2022). N6-methyladenosine RNA methylation regulator-related alternative splicing gene signature as prognostic predictor and in immune microenvironment characterization of patients with low-grade glioma. Front. Genet..

[27] Meng S, Liu Y, Wang X, Wu X, Xie W, Kang X, Liu X, Guo L, Wang C (2022). The prognostic value and biological significance of gap junction beta protein 2 (GJB2 or Cx26) in cervical cancer. Front. Oncol..

[28] Lin Z, Wang R, Huang C, He H, Ouyang C, Li H, Zhong Z, Guo J, Chen X, Yang C (2022). Identification of an immune-related prognostic risk model in glioblastoma. Front. Genet..

[29] Xu JL (2022). Wilms tumor 1-associated protein expression is linked to a T-cell-inflamed phenotype in pancreatic cancer. Dig. Dis. Sci..

[30] Wei H, Chen F, Chen J, Lin H, Wang S, Wang Y, Wu C, Lin J, Zhong G (2022). Mesenchymal stem cell derived exosomes as nanodrug carrier of doxorubicin for targeted osteosarcoma therapy via SDF1-CXCR4 axis. Int. J. Nanomed..

[31] Cheng J, Zhang Y, Wan R, Zhou J, Wu X, Fan Q, He J, Tan W, Deng Y (2022). CEMIP promotes osteosarcoma progression and metastasis through activating notch signaling pathway. Front. Oncol..

[32] Sánchez-Sánchez AM, Turos-Cabal M, Puente-Moncada N, Herrera F, Rodríguez C, Martín V (2022). Calcium acts as a central player in melatonin antitumor activity in sarcoma cells. Cell. Oncol..

[33] Zheng S, Liu Y, Sun H, Jia J, Wu T, Ding R, Cheng X (2021). Identification of abnormally high expression of POGZ as a new biomarker associated with a poor prognosis in osteosarcoma. Eur. J. Histochem..

[34] Fan L, Peng C, Zhu X, Liang Y, Xu T, Xu P, Wu S (2022). Dihydrotanshinone I enhances cell adhesion and inhibits cell migration in osteosarcoma U-2 OS cells through CD44 and chemokine signaling. Molecules.

[35] Lv B, Gao G, Guo Y, Zhang Z, Liu R, Dai Z, Ju C, Liang Y, Tang X, Tang M (2021). Serglycin promotes proliferation, migration, and invasion via the JAK/STAT signaling pathway in osteosarcoma. Aging.

[36] Assi T, Watson S, Samra B, Rassy E, Le Cesne A, Italiano A, Mir O (2021). Targeting the VEGF pathway in osteosarcoma. Cells.

[37] Gul Mohammad A, Li D, He R, Lei X, Mao L, Zhang B, Zhong X, Yin Z, Cao W, Zhang W (2022). Integrated analyses of an RNA binding protein-based signature related to tumor immune microenvironment and candidate drugs in osteosarcoma. Am. J. Transl. Res..

[38] Niu J, Yan T, Guo W, Wang W, Zhao Z, Ren T, Huang Y, Zhang H, Yu Y, Liang X (2020). Identification of potential therapeutic targets and immune cell infiltration characteristics in osteosarcoma using bioinformatics strategy. Front. Oncol..

[39] Wang J, Gong M, Xiong Z, Zhao Y, Xing D (2022). ADAM19 and TUBB1 correlates with tumor infiltrating immune cells and predicts prognosis in osteosarcoma. Comb. Chem. High Throughput Screen..

[40] Ma Y, Tong C, Xu M, He H, Chen C (2022). Bioinformatics analysis reveals an association between autophagy, prognosis, tumor microenvironment, and immunotherapy in osteosarcoma. J. Oncol..

[41] He Y, Zhou H, Xu H, You H, Cheng H (2022). Construction of an immune-related lncRNA signature that predicts prognosis and immune microenvironment in osteosarcoma patients. Front. Oncol..

[42] Wang Q, Chen Y, Gao W, Feng H, Zhang B, Wang H, Lu H, Tan Y, Dong Y, Xu M (2022). Identification and validation of a four-gene ferroptosis signature for predicting overall survival of lung squamous cell carcinoma. Front. Oncol..

[43] Zhou X, Yao L, Zhou X, Cong R, Luan J, Wei X, Zhang X, Song N (2022). Pyroptosis-related lncRNA prognostic model for renal cancer contributes to immunodiagnosis and immunotherapy. Front. Oncol..

[44] Huang L, Zhang L, Chen X (2022). Updated review of advances in microRNAs and complex diseases: Taxonomy, trends and challenges of computational models. Brief. Bioinform..

[45] Huang L, Zhang L, Chen X (2022). Updated review of advances in microRNAs and complex diseases: Towards systematic evaluation of computational models. Brief. Bioinform..

[46] Huang L, Zhang L, Chen X (2022). Updated review of advances in microRNAs and complex diseases: Experimental results, databases, webservers and data fusion. Brief. Bioinform..

[47] Wang SH, Zhao Y, Wang CC, Chu F, Miao LY, Zhang L, Zhuo L, Chen X (2024). RFEM: A framework for essential microRNA identification in mice based on rotation forest and multiple feature fusion. Comput. Biol. Med..

