# Supplementary figures and images for: PDE1B, a potential biomarker associated with tumor microenvironment and clinical prognostic significance in osteosarcoma
Source: Sci Rep. 2024 Jun 14;14:13790. doi: 10.1038/s41598-024-64627-y (PMC11178771; doi:10.1038/s41598-024-64627-y)

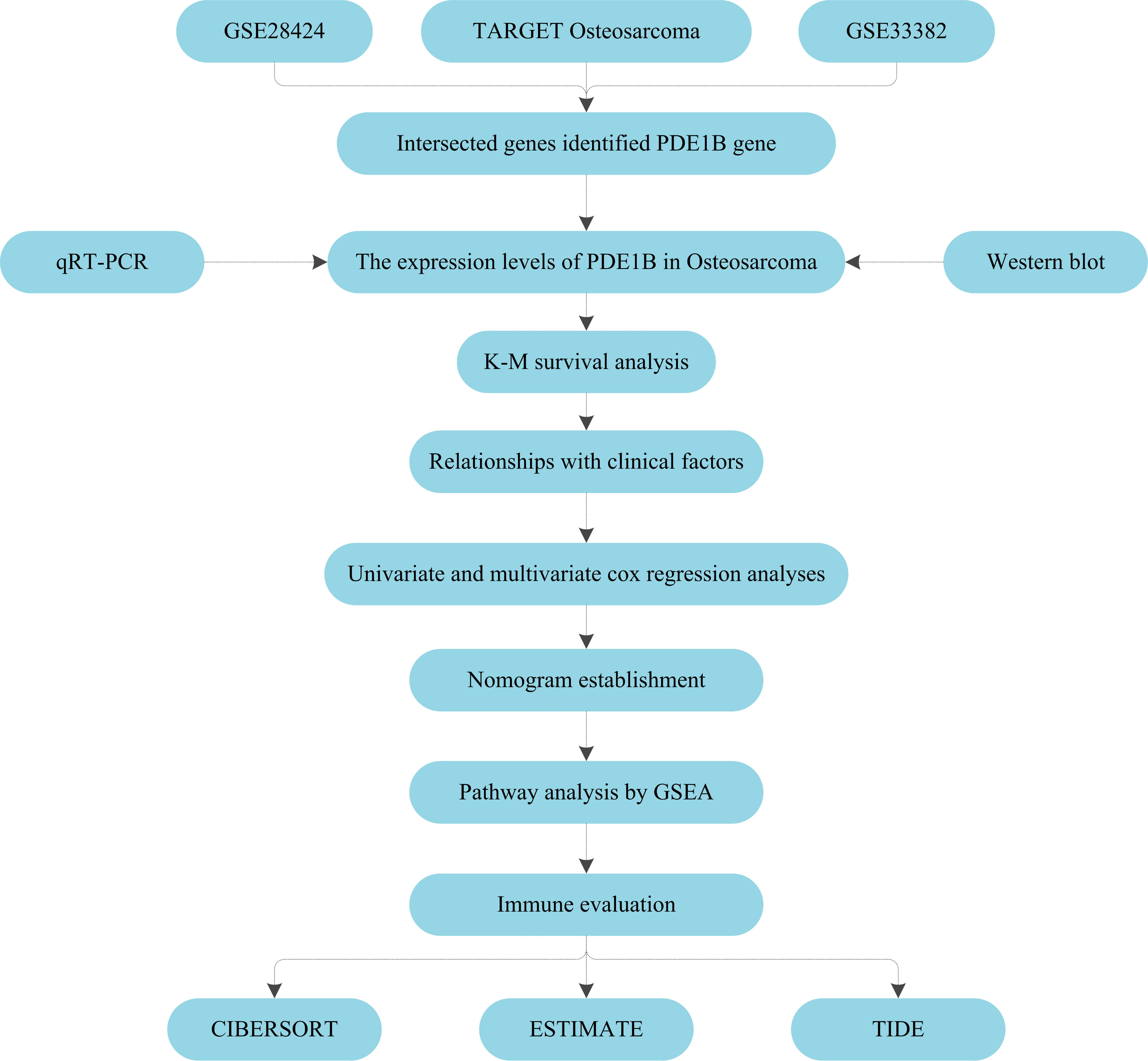

Supplement: Supplementary file 1 — Supplementary Information 1. [file 41598_2024_64627_MOESM1_ESM.tif]
